# Accuracy, reliability, and timing of visual evaluations of decay in fresh-cut lettuce

**DOI:** 10.1371/journal.pone.0194635

**Published:** 2018-04-17

**Authors:** Ivan Simko, Ryan J. Hayes

**Affiliations:** 1 U.S. Department of Agriculture, Agricultural Research Service, U.S. Agricultural Research Station, Crop Improvement and Protection Research Unit, Salinas, California, United States of America; 2 U.S. Department of Agriculture, National Forage Seed Production Research Center, Corvallis, Oregon, United States of America; Wageningen University, NETHERLANDS

## Abstract

Visual assessments are used for evaluating the quality of food products, such as fresh-cut lettuce packaged in bags with modified atmosphere. We have compared the accuracy and the reliability of visual evaluations of decay on fresh-cut lettuce performed with experienced and inexperienced raters. In addition, we have analyzed decay data from over 4.5 thousand bags to determine the optimum timing for evaluations to detect differences among accessions. Lin’s concordance coefficient (*ρ*_*c*_) that takes into consideration both the closeness of the data and the conformance to the identity line showed high repeatability (intra-rater reliability, *ρ*_*c*_ = 0.97), reproducibility (inter-rater reliability, *ρ*_*c*_ = 0.92), and accuracy (*ρ*_*c*_ = 0.96) for experienced raters. Inexperienced raters did not perform as well and their ratings showed decreased repeatability (*ρ*_*c*_ = 0.93), but even larger reduction in reproducibility (*ρ*_*c*_ = 0.80) and accuracy (*ρ*_*c*_ = 0.90). We have detected that 5.3% of ratings were outside of the 95% limits of agreement. These under- or overestimates were predominantly found for bags with intermediate levels of decay, which corresponds to the middle of the rating scale. This occurs because intermediate amounts of decay are more difficult to discriminate than extremes. The frequencies of aberrant ratings for experienced raters ranged from 0.6% to 4.4% (mean = 2.1%), for inexperienced raters the frequencies were substantially higher, ranging from 6.1% to 15.6% (mean = 9.4%). Therefore, we recommend that new raters receive training that includes practical examples in this range of decay, use of standard area diagrams, and continuing interaction with experienced raters (consultation during actual rating). Very high agreement among experienced raters indicate that visual ratings can be successfully used for evaluations of decay, until a more objective, rapid, and affordable method is developed. We recommend evaluating samples at multiple time points until 42 days after processing (about 80% decay on average) and then combining these individual ratings into the area under the decay progress stairs (AUDePS) score. Applying this approach, experienced evaluators can accurately detect difference among lettuce accessions and identify lettuce cultivars with reduced decay.

## Introduction

Fresh-cut lettuce packaged in salad mixes is a desirable product due to its convenience for consumers [1]. Production in the U.S. increased dramatically during the 1990s, experiencing a more than six-fold increase in value [1]. While there are no current estimates of the retail value of fresh-cut lettuce, the segment represents 25–35% of raw product production in Monterey county of California, which has a farm gate value of approximately 1.5 billion dollars [2]. Most fresh-cut products involve harvesting whole mature heads of romaine or iceberg type lettuce, cutting the leaves to a specified size, mixing with other vegetables, and packaging the salad in clear specialized films with modified atmospheres (modified atmosphere packaging, MAP) [3, 4]. Harvesting, handling and packaging technologies for fresh-cut lettuce have continually evolved as the market has expanded. However, the cultivars used to make packaged salads have generally been bred using the same approaches as those used to breed cultivars for marketing as whole heads.

Breeding lettuce cultivars specifically suited for fresh-cut processing can improve the efficiency of production and the quality of the product [5]. Targets for genetic improvement include pre-processing characters such altered plant architecture, leaf color, slow bolting, and freedom from internal defects (e.g. tipburn). Post-processing traits are increasingly important in breeding programs, since the cutting involved can lead to pink or brown discoloration through wound-induced oxidation and shorten lettuce shelf-life through decay or deterioration of the leaf pieces. Breeding for reduced discoloration has been conducted [5], though the wide spread use of MAP by commercial processors in the U.S. results in generally acceptable control of this problem [3]. Decay of lettuce pieces, seen as darkening, water logging, and deterioration, will eventually still occur in MAP and causes the end of the salad’s shelf-life. Decay of salad in MAP is a heritable trait of lettuce conditioned by both small and large effect quantitative trait loci (QTL) [6, 7]. As a result, the rate of decay can be manipulated through selective breeding and lettuce germplasm has been released that was selected or evaluated for the rate of decay as part of the breeding procedure [8–12].

Improved genotypes that become successful cultivars arise from a rare combination of numerous genes and the evaluation of large breeding populations is necessary to discover these genotypes. The likelihood of success in a plant breeding program can be improved by developing assessment techniques that are fast and accurate, thereby enabling the efficient assessment of large populations. Pilot scale techniques for making salad in MAP that simulate commercial processing can be developed for shelf-life experiments, and with simple modifications allow processing of thousands of MAP bags of a breeding population within a few days. Assessment of lettuce decay in MAP is a critical step in these experiments and several approaches were previously used. They can be categorized as either destructive, that can be used for only a single evaluation, or non-destructive, that allow multiple evaluations of the same sample. Two destructive approaches (manual sorting of leaf pieces and tissue conductivity) and three non-destructive approaches (hyperspectral imaging, chlorophyll fluorescence imaging, and visual observations) were previously tested in our laboratory [6, 13–15].

Opening MAP bags with fresh-cut lettuce and manually sorting leaf pieces into categories provides a good estimate of tissue decay. However, the process is very slow and somewhat subjective. For more rapid evaluations, a piece of a leaf may be considered decayed when any portion of the piece is decayed [13]. This approach speeds up the sorting process, but likely leads to an overestimate of the percent of decayed tissue. Electrolyte leakage from decayed cells can be measured by the means of electric conductivity [15]. This approach, though somewhat faster than manual sorting, is still very slow. Also, conductivity may not always provide an exact estimate of decay, as decayed tissue may release a different amount of cell lysate; e.g. cultivars may differ for total potential leakage and waterlogged tissue with relatively intact cell walls releases less electrolyte than a tissue with completely ruptured cells.

Non-destructive evaluation of decay in fresh-cut lettuce was successfully performed with optical sensors using hyperspectral imaging and chlorophyll fluorescence imaging [15]. These approaches, if automated, could allow for very fast evaluations directly in MAP bags. Their disadvantage is a substantial initial cost for setting up such automated systems. Also, for the systems to function properly, it is important to correctly calibrate sensors and determine thresholds for distinguishing fresh and decayed tissue. Another non-destructive, yet very simple approach is a visual evaluation of decay directly in MAP bags. Similarly to the approaches that use optical sensors, this evaluation is performed only on the lettuce pieces visible through the transparent plastic film, thus considering only a partial sample of processed tissue. We have previously employed this approach using 0–5 [13] and 0–10 [6, 14] linear rating scales, and shown that visual evaluations are strongly correlated with the methods that use manual sorting of leaf pieces into categories, electric conductivity, and optical sensors (hyperspectral or chlorophyll fluorescence imaging) [15]. A general problem with visual evaluations may be subjectivity, particularly if ratings are performed by inexperienced raters [16–18]. Therefore, we have investigated both accuracy and reliability of the visual rating systems, and compared evaluations performed by experienced- and inexperienced raters. Reliability measures the extent to which repeated assessments on the same MAP bags yield similar results [19]. Intra-rater reliability (repeatability) is the closeness between ratings performed by the same rater, while inter-rater reliability (reproducibility) is the closeness between ratings performed by different raters. Accuracy is defined as the degree of conformity between visual ratings and some recognized standard value that is presumed to be close to the true treatment value [19].

Initially, all tissue in MAP bags have no decay resulting in no differences between treatments (accessions, cultivars, families, etc.). Treatment differences increase during storage, as some genotypes decay while others do not. As all MAP bags begin to exhibit extensive decay, treatment differences again become smaller. Since large treatment differences are easier to detect, the timing of fresh-cut lettuce evaluations is important. In our previous studies, we mostly performed evaluations in weekly intervals, starting usually one week after processing [6, 14, 15]. Because we studied dynamics of the whole decay process, not just the early stages of decay, the evaluations generally lasted until all (or almost all) of the material was completely decayed. The wealth of these data (several tens of thousands of MAP bags) can be used to determine the time when the differences between samples from different accessions are the most pronounced. Such information can be helpful to other researchers who are looking for the optimal evaluation time-points.

The objectives of the current study were to compare the accuracy and the reliability of visual evaluations of decay on fresh-cut lettuce performed with experienced and inexperienced raters, and to determine the optimum timing for evaluations to detect differences among accessions.

## Material and methods

### Lettuce cultivation and processing

Lettuce plants were grown under field conditions typical for lettuce production in the Salinas Valley of California, USA [20, 21]. Lettuce heads were harvested at market maturity and processed one or two days after harvest. Before processing, the harvested heads were kept in cold storage at 3.5°C. Processing of heads for fresh-cut salad was done as previously described [6, 13, 14]. The lettuce cores (i.e. stems) were removed from heads, and the remaining leaves were cut into pieces of about 2.5 cm^2^. Subsequently, the salad was washed with 0.0016 mol l^-1^ NaOCl, dried in a commercial salad centrifuge, and placed into transparent polyethylene bags. Each plastic bag (22.8 cm × 30.5 cm) contained 340g of tissue. Bags were flushed three times with N_2_, sealed, and stored in the dark at 3.5°C. The N_2_ flushing has been recommended as the most effective and economical approach when establishing an initial low oxygen atmosphere within the packaging, thus reducing enzymatic browning reactions and pinking [22]. Flushing polyethylene bags that contained fresh-cut lettuce with N_2_ brings down the O_2_ level to about 1.5% as was determined on a random sample of 50 bags using PBI Dansensor CheckMate 9900 meter (PBI-Dansensor, Ringsted, Denmark). Five to nine bags were processed per accession in each of the analyzed experiments. Bags were considered to be assigned at random (completely randomized design). Visual rating of decay in MAP bags was performed by experienced raters. Processing and storing of samples was performed according to the approach developed in consultation with the lettuce industry. More detailed information about processing can be found in our previous publications [6, 13, 14].

### Evaluation of decay

The type of lettuce decay evaluated in this study is predominantly caused by genetic determinants [6] and is not substantially affected by concentrations of oxygen (O_2_) or carbon dioxide (CO_2_) [6, 13] in MAP. Evaluations of decay were performed in weekly intervals, starting one week after processing and continuing until all (or almost all) bags with lettuce showed complete decay. Evaluation of decay was performed on a 0 through 10 scale that corresponds to the estimated percentage of decayed tissue divided by 10 and rounded to the nearest whole number. In one of the experiments, 90 bags with a range of decay were randomly selected three weeks after processing from all evaluated bags. This subset of bags was used to compare evaluations performed by five experienced and four inexperienced raters. This subset of bags was randomly rated twice by each rater without a time constraint. Raters who previously evaluated bags on at least 10 occasions were considered to be experienced; while inexperienced raters previously did not evaluate more than two experiments (a brief training was provided to the new raters). Decay was recognized as the presence of water-soaked tissue. No other blemishes on tissue, such as oxidative browning, pinking, or tipburn were considered in these evaluations. Ratings performed by inexperienced raters were used for analyses of accuracy and reliability, but were eliminated from analyses of timing where only ratings of experienced raters were considered to ensure high uniformity.

### Collection of data

**Fig 1:**
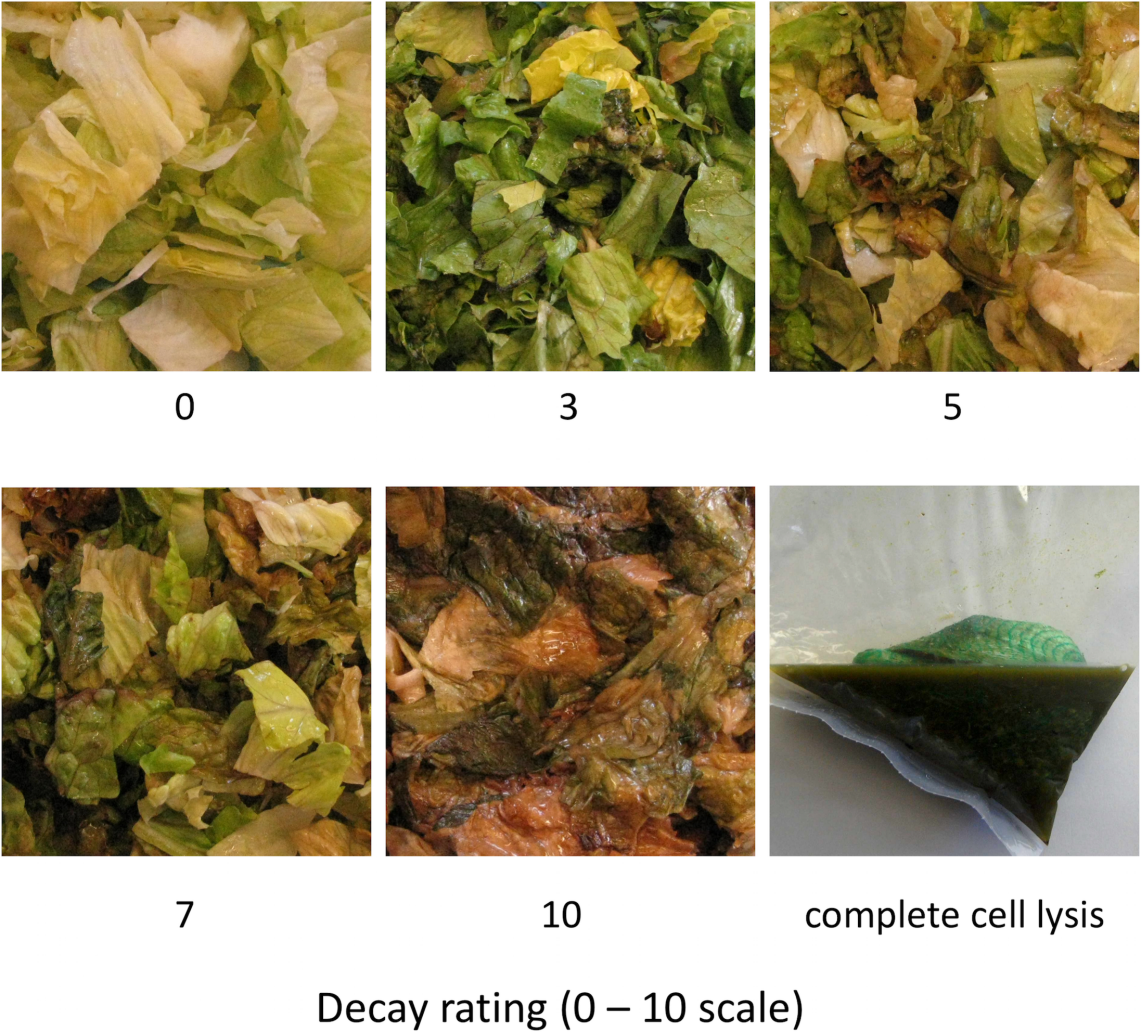
Differences in decay of fresh-cut lettuce stored in modified atmosphere packages (MAP) at 3.5°C. The decay rating on the 0 to 10 scale corresponds to the estimated percentage of decayed tissue divided by ten. Note that the tissue samples were removed from MAP bags before photographing them. At times, decay can be accompanied by a profound cell lysis. The photograph at the lower-right corner shows the tissue sample with most or all of the cells already disrupted. This sample was photographed while still inside the MAP bag; therefore, a plastic mesh bag can be seen above the cell lysate. The mesh bag was used to keep fresh-cut tissue together during sample preparation.

Decay progress data were collected from 4,535 bags (including 90 bags described in the previous paragraph) evaluated in eight independent experiments. These eight experiments were selected for statistical analyses because comprehensive data were amassed for dynamics of the whole decay process (Fig 1). Statistical analyses were performed on weekly ratings of decay, but also on the area under the decay progress stairs (AUDePS) [14] values that combine weekly ratings into a single index value [23]. Data from weekly ratings were also used to determine time (measured in days) to 100% decay (T100D) for each bag [6]. Moreover, we also calculated time needed to reach 10% decay through time needed for 90% decay (T10D, T20D, … T80D, T90D) and used these data for additional statistical analyses.

### Statistical analyses

**Fig 2:**
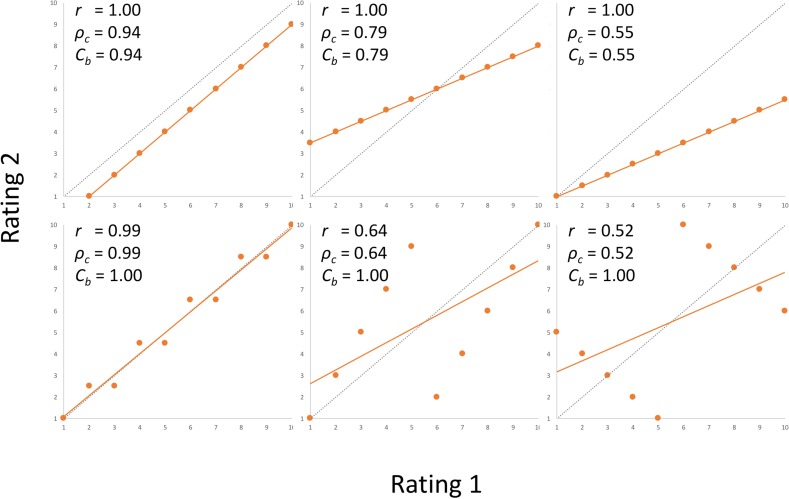
Examples of indexes used for evaluations of agreement between pairs of ratings. Pearson correlation coefficient (*r*) provides good information about the closeness of the ratings to the best-fitting line (best fitting line is in orange). Lin’s concordance coefficient (*ρ*_*c*_) combines both the closeness of the ratings to the best-fitting line and how the best-fitting line conforms to the identity line. Identity line (also called line of equality, 1:1 line, or x = y line) leads from the origin at 45-degrees (slope of 1) and represents the perfect agreement between evaluations (identity line is in dashed black). Coefficient of bias (*C*_*b*_) represents the ratio between the concordance coefficient and the correlation coefficient *C*_*b*_ = *ρ*_*c*_ /*r*. Upper row shows pairs of ratings with perfect correlation coefficient, but imperfect concordance coefficient. Lower row shows pairs of ratings with coefficient of bias = 1, but imperfect concordance coefficient.

Accuracy, reliability, and bias of visual ratings were determined from the assessments performed on 90 MAP bags that were evaluated twice by nine raters. Intra-rater reliability (repeatability) and inter-rater reliability (reproducibility) indices were determined from both Pearson correlation coefficient (*r*) and Lin’s concordance coefficient (*ρ*_*c*_) [24] that were calculated from the respective pairs of ratings of each bag. Though the index based on Pearson correlation coefficient provides good information about closeness of the ratings to the best-fitting line, it was criticized [25–27] because this index does not take into the consideration how the best-fitting line conforms to the identity line (Fig 2, upper row) (identity line represents the perfect agreement between evaluations). Therefore, Lin’s concordance coefficient (*ρ*_*c*_) [24] that combines both the closeness of the data and the conformance to the identity line, is preferred for evaluations of reliability. Coefficient of bias (*C*_*b*_) that estimates deviation between the best-fitting line and the identity line (Fig 2, lower row) can be calculated as: *C*_*b*_ = 2/(*v*+1/*ν*+*u*^2^), where *ν* = *σ*_1_/*σ*_2_, and *u* = (μ_1_ - μ_2_) / (*σ*_1_ × *σ*_2_)^-2^. The terms μ_1_, μ_2_ and *σ*_1_, *σ*_2_ are the means and the standard deviations for the two data sets [17]. The relationship between Lin’s concordance coefficient, Pearson correlation coefficient, and coefficient of bias is defined as: *ρ*_*c*_ = *r* × *C*_*b*_ [17]. In each of these three statistics, higher values indicate a better match, with value of 1 being the maximum.

In the absence of a universal gold standard for rating of decay, we used a composite reference standard (CRS) for assessing the accuracy of ratings [28]. In this procedure, each rating is interpreted in the context of all other ratings that are used to calculate the CRS. In our study, CRS was calculated as a mean of all ratings excluding the two ratings of the rater being analyzed. Because of this, each rater was compared to a slightly different CRS. Accuracy of the rating was thus defined as the agreement between the rating and the CRS, and determined from Lin’s concordance. For comparison, we also calculated Pearson correlation coefficient, and coefficient of bias for the same data.

Values of *r* were transformed using Fisher’s *r*–to—*z* transformation prior to statistical analyses and back-transformed after the analyses. Differences in the performance of experienced and inexperienced raters (*r*, *ρ*_*c*_, and *C*_*b*_ values) were evaluated with *t*-tests. Principal component analysis was performed on rating data to illustrate relationships among individual assessments and raters. A Bland-Altman plot [29] was constructed to allow visual inspection of differences in ratings and to identify possible systematic bias. We used a modification of the Bland-Altman approach that recommends plotting differences against the gold standard [30], in our case, against CRS.

The optimal evaluation time point for detecting the most significant difference among accessions was determined using decay progress data on 4,535 bags from eight experiments. Statistical analyses were performed on weekly ratings, on AUDePS values, and on time needed for samples to reach a certain percentage of decay (T10D to T100D). Because data from weekly ratings were not always normally distributed, non-parametric Kruskal-Wallis (KW) test was used for data analysis. We compared H-statistics from KW tests to find the time when difference among accessions were most significant.

Pearson correlation coefficient (*r*), *t*-test, principal components analysis, and Kruskal-Wallis test were calculated in JMP software v. 11.1.1 (SAS Institute, Cary, NC). Calculations for coefficient of bias (*C*_*b*_) and for 95% limits of Bland-Altman plot were done in Microsoft Excel v.15.30 (Microsoft, Redmond, WA, USA). Lin’s concordance coefficient (*ρ*_*c*_) was calculated with the online calculator provided by the New Zealand National Institute of Water and Atmospheric Research (https://www.niwa.co.nz/node/104318/concordance).

## Results and discussion

### Accuracy and reliability of visual evaluations

**Fig 3:**
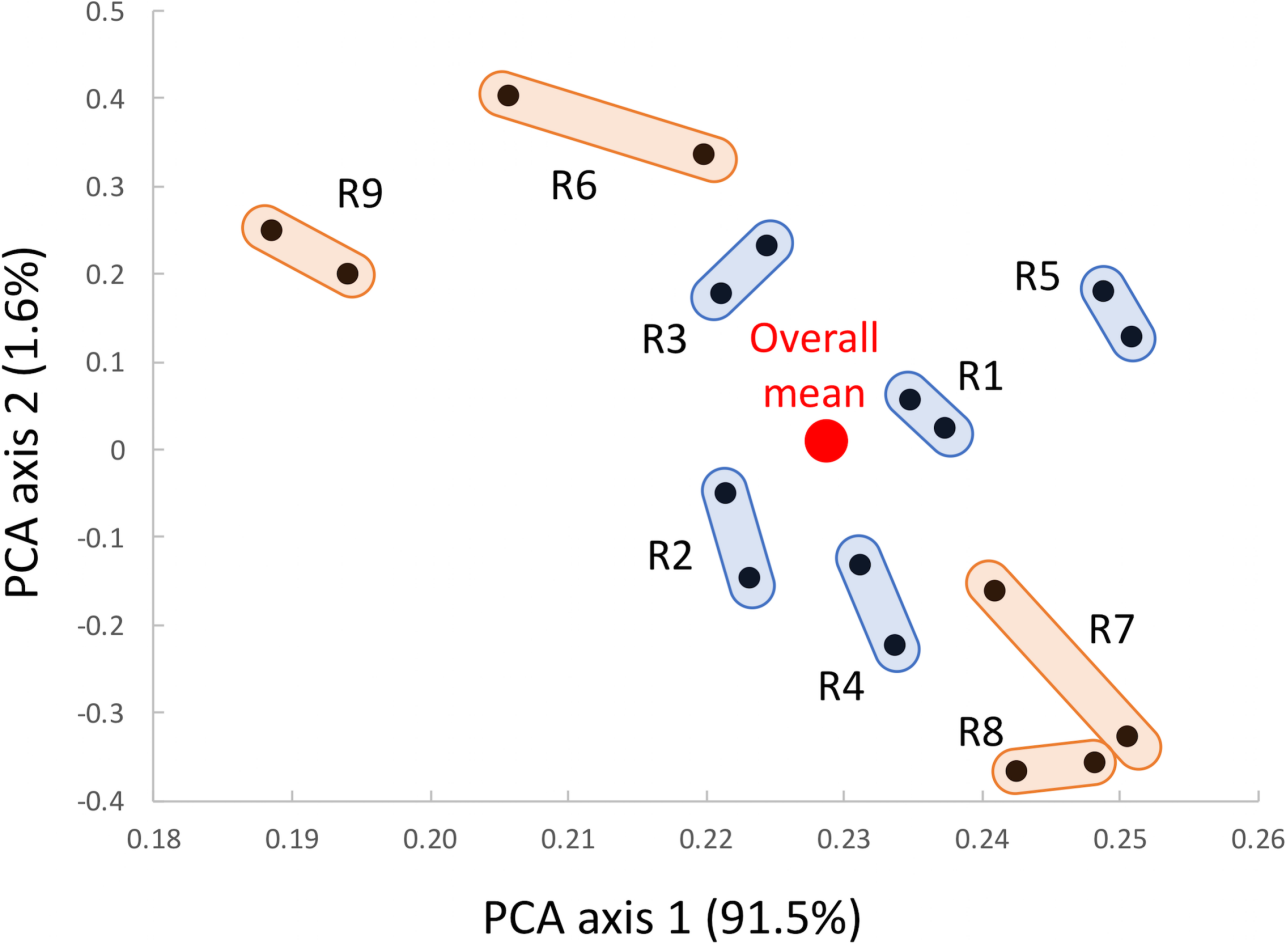
Results of principal component analysis performed on visual ratings. Decay of fresh-cut lettuce was evaluated in 90 MAP bags by nine raters; each rater evaluating the set of bags twice. R1 to R5 were experienced raters (blue color), while R6 to R9 were inexperienced raters (orange color). A pair of ratings from the same rater are connected by obrounds. The overall mean from all ratings is indicated by the red circle.

**Fig 4:**
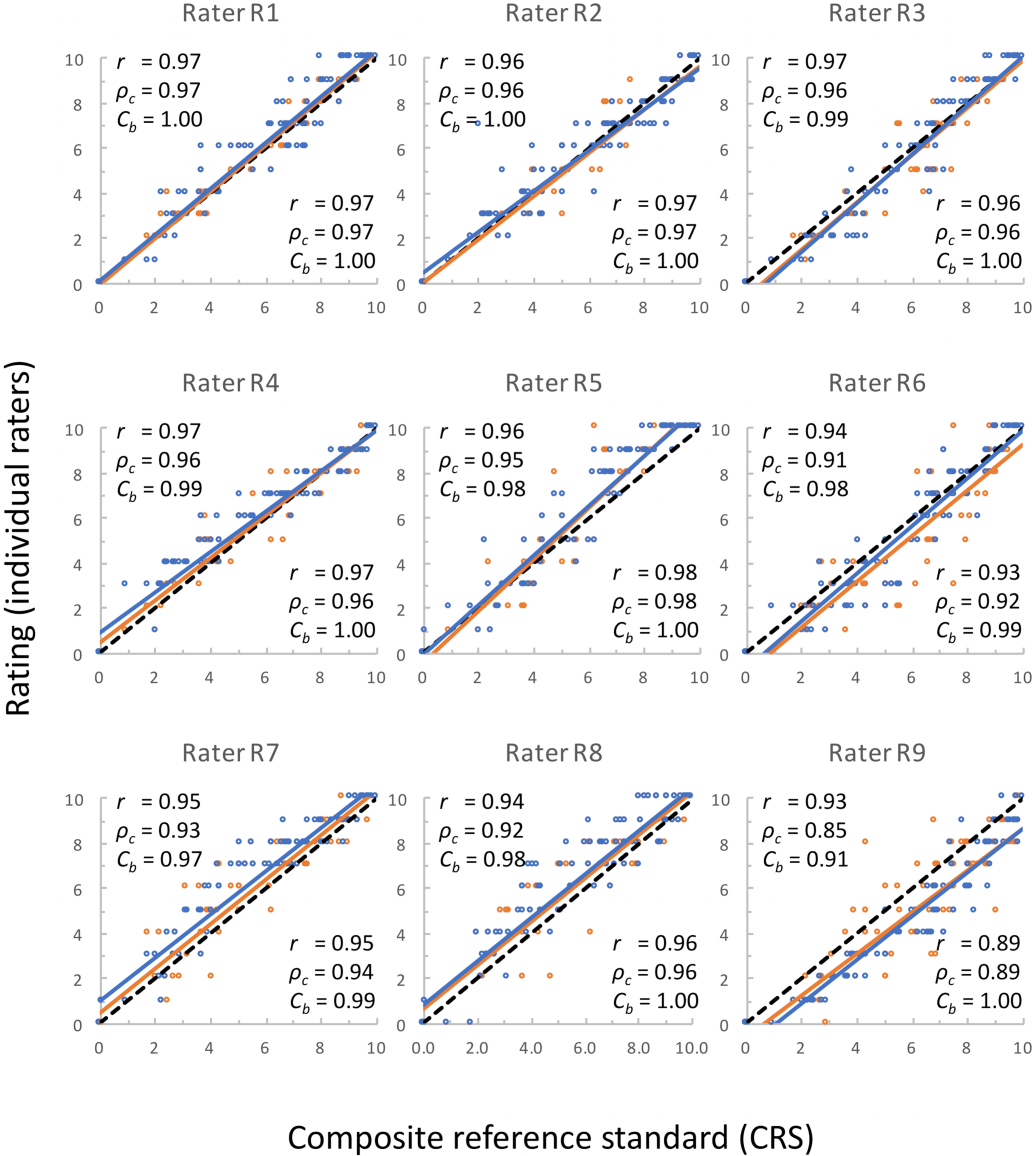
Relationship between the composite reference standard (CRS) and 18 individual ratings performed by nine raters. Orange and blue lines show the best-fit between CRS and two sets of ratings. Black dashed lines are identity lines that show the perfect agreement between CRS and individual ratings. Within each panel, values in the lower right corner show Pearson correlation coefficient (*r*), Lin’s concordance coefficient (*ρ*_*c*_), and coefficient of bias (*C*_*b*_) between two independent ratings of the same rater (measurement of repeatability, or intra-rater reliability). Values in the upper left corners show the same coefficients but between CRS and individual raters (measurements of accuracy). Accuracy of a rater is the mean of accuracies calculated for two ratings of the rater. R1 to R5 were experienced raters, while R6 to R9 were inexperienced raters.

Ratings of 90 bags by nine raters indicated that decay of tissue in the analyzed MAP bags ranged from 0 to 10. Principal components analysis showed that two ratings from the same rater were usually more similar to each other, than to ratings from other raters (Fig 3). However, occasionally, ratings from different raters showed similarity that was higher than that within a rater (e.g. raters R7 and R8). Ratings of experienced raters were more closely clustered together and to the overall mean than those from inexperienced raters (Fig 3). Intra-rater correlation (*r*) ranged from 0.89 to 0.98 (mean of 0.95), and coefficient of bias (*C*_*b*_) from 0.99 to 1.00 (mean of 1.00). Concordance coefficient (*ρ*_*c*_) that is the best indicator of intra-rater reliability (repeatability) ranged from 0.89 to 0.98 (mean of 0.95) (Fig 4). Accuracy, that was measured as concordance (*ρ*_*c*_) between individual ratings and CRS, ranged from 0.85 to 0.98 (mean = 0.93). For the same pairs of ratings, correlation coefficient ranged from 0.91 to 0.98 (mean = 0.95) and coefficient of bias from 0.90 to 1.00 (mean = 0.98) (Fig 4). These data show generally good agreement in ratings, with the exception of the rater R9 that had the lowest *r* and *ρ*_*c*_ values for both repeatability (0.89) and accuracy (0.93 and 0.85, respectively). This rater, however, was evaluating lettuce decay for the first time. Similar results were obtained when the percentage of diseased tissue was visually assessed on plant leaves or fruits [16–18, 31]. In these studies, intra-rater reliability was estimated with Pearson correlation coefficient (*r*) and averaged 0.97 [18], 0.96 [16], 0.95 [17], and 0.94 [31]. Accuracy of visual observations based on concordance coefficient (*ρ*_*c*_) was 0.94 (for experienced raters) [16], 0.89 [18], and 0.86 [17].

We detected a substantial difference in repeatability, reproducibility, and accuracy of ratings between experienced and inexperienced raters. Experienced raters had the average repeatability of *ρ*_*c*_ = 0.97, while the value for inexperienced raters was *ρ*_*c*_ = 0.93 (Table 1). Though this difference is not very large in absolute terms, it was significant at *p* < 0.05. More significant (*p* < 0.001) and larger difference was observed in reproducibility that increased from *ρ*_*c*_ = 0.80 for inexperienced raters to *ρ*_*c*_ = 0.92 for experienced raters. With experience, the accuracy of ratings increased from *ρ*_*c*_ = 0.90 to *ρ*_*c*_ = 0.96 (*p* < 0.001). Similar *p*-values for differences between experienced and inexperienced raters were detected also when correlation coefficient (*r*) and coefficient of bias (*C*_*b*_) were compared (Table 1). The effect of a rater’s experience on visual assessments is well known. It was reported before that the accuracy of disease ratings increased with experience from *ρ*_*c*_ = 0.69 to *ρ*_*c*_ = 0.86 [18]. Use of standard area diagrams (SAD) increased accuracy in both groups, particularly in the less experienced one (*ρ*_*c*_ = 0.88 for inexperienced raters, *ρ*_*c*_ = 0.89 for experienced raters). Not only experience, but also initial training significantly improves repeatability and reproducibility of visual ratings. Concordance coefficient (*ρ*_*c*_) for repeatability (intra-rater reliability) of percentage of leaf area with disease symptoms increased from 0.76 before training to 0.96 after training and additional instructions [16]. For the same group or raters, reproducibility (inter-rater reliability) of visual assessments increased from *ρ*_*c*_ = 0.26 before training to *ρ*_*c*_ = 0.85 after training and instructions. Previous results and current data show the importance of appropriate training, but also additional interaction between experienced and inexperienced raters for good agreements of ratings. For our current study, it is important to note that repeatability of ratings was generally very good even for inexperienced raters (*ρ*_*c*_ from 0.89 to 0.96). It is accuracy of ratings, however, that needs to be improved (Figs 3 and 4). This can be done with proper training [32].

**Table 1:** Effect of rater’s experience on accuracy and reliability of visual ratings.

Parameter	Intra-rater reliability (repeatability)				Inter-rater reliability (reproducibility)				Accuracy			
	E.R. (95% CI)^a^	I.R. (95% CI)^a^	E.R–I.R. Diff.	*P*-value	E.R. (95% CI)^a^	I.R. (95% CI)^a^	E.R–I.R. Diff.	*P*-value	E.R. (95% CI)^a^	I.R. (95% CI)^a^	E.R–I.R. Diff.	*P*-value
Coefficient of bias (*C*_*b*_)	0.999 (0.998–1.000)	0.994 (0.990–0.997)	0.005	0.015	0.987 (0.984–0.991)	0.910 (0.881–0.939)	0.077	< 0.001	0.994 (0.990–0.998)	0.960 (0.938–0.983)	0.034	0.005
Pearson correlation coefficient (*r*)	0.969 (0.962–0.976)	0.931 (0.901–0.961)	0.028	0.038	0.931 (0.927–0.934)	0.872 (0.861–0.884)	0.058	< 0.001	0.966 (0.961–0.970)	0.939 (0.927–0.951)	0.027	< 0.001
Lin’s concordance coefficient (*ρ*_*c*_)	0.968 (0.961–0.974)	0.925 (0.895–0.955)	0.043	0.017	0.919 (0.913–0.925)	0.795 (0.765–0.824)	0.124	< 0.001	0.960 (0.954–0.966)	0.902 (0.876–0.927)	0.058	< 0.001

^a^ Mean value and 95% confidence intervals

E.R.–experienced raters. I.R.–inexperienced raters

**Fig 5:**
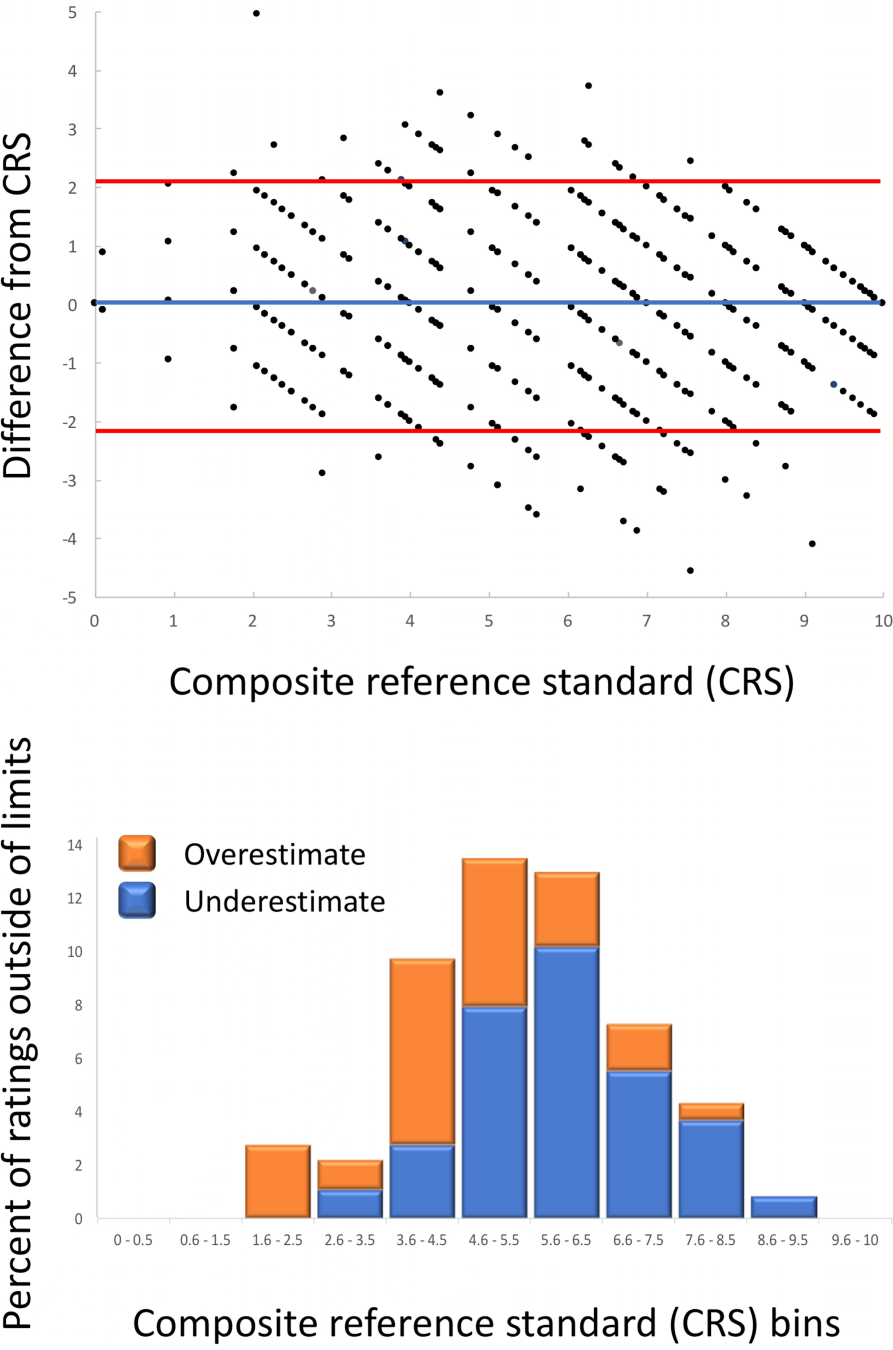
Difference between composite reference standard (CRS) and 18 individual ratings performed by nine raters on the set of 90 MAP bags. Upper panel shows a Bland-Altman plot. Blue line indicates the mean difference between CRS and ratings. Orange lines show the upper and the lower values for the 95% limit of agreement. Lower panel shows percentage of ratings for the particular CRS bin that are outside of the 95% limit of agreement. Orange bars show frequency of overestimates, while blue bars show frequency of underestimates.

To determine if systematic bias in ratings of decay exists, we used a Bland-Altman difference plot that provides an insight into the agreement between CRS and individual ratings (Fig 5, upper panel). We have detected that 5.3% of ratings were outside of the 95% limits of agreement. These ratings were predominantly found around the middle of the rating scale (3.6 to 7.5 CRS) because differences in decay are more difficult to discriminate in the middle of the scale than at the extremes. Similar pattern in visual assessment is well known in plant pathology, where the scale was designed to compensate for this type of human error [33]. Most of the ratings that were outside of the 95% limits in the earlier stages of decay (CRS 1.6 to 4.5) overestimated the decay, while those that were outside of the limits at the later stages of decay (CRS 5.6 to 9.5) underestimated the decay (Fig 5, lower panel). Again, these observations are in agreement with those reported in plant pathology where raters overestimate the percentage of chlorotic and necrotic leaf area at low severity [34]. A number of factors were suggested that may influence visual rating of disease severity [35]. Some of these factors may apply for the rating of lettuce decay in MAP bags, such as rater intrinsic ability, value preferences by raters, number and size of decayed pieces, leaf structure and color, complexity of symptoms, and interaction among multiple factors. Other subjective sensory evaluations performed on growing lettuce plants or processed tissue showed previously good match with objective measurements; e.g., red and green leaf color and spectral analyses [36], tissue decay and chlorophyll fluorescence imaging or hyperspectral imaging [15], leaf surface browning and colorimetric measurement [12], and off-odor development and ethanol accumulation [12]. However, neither one of these studies analyzed samples multiple times to determine repeatability (intra-rater reliability) of results or compared performance of multiple raters to determine reproducibility (inter-rater reliability) of sensory evaluations.

**Fig 6:**
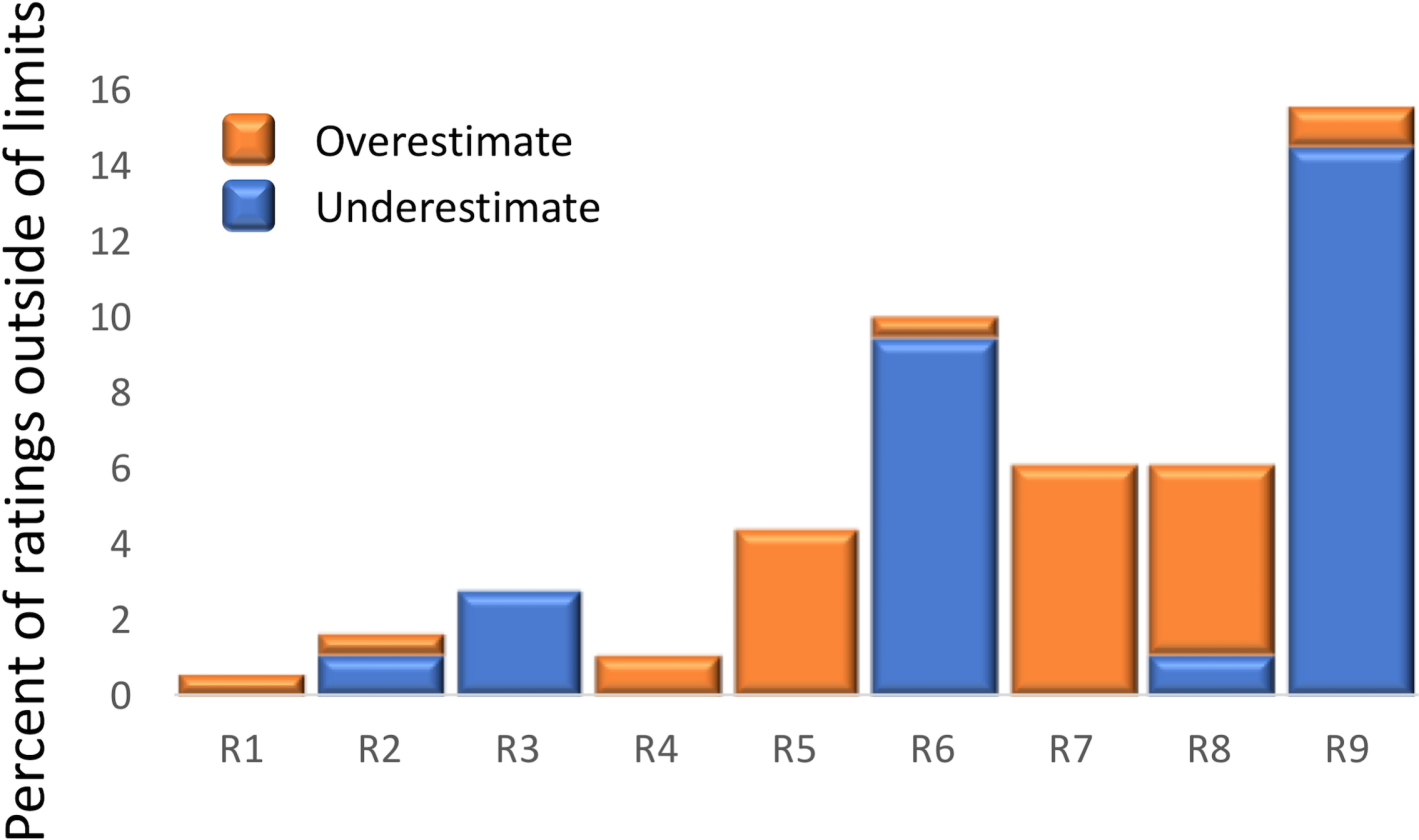
Percentage of ratings for individual raters that are outside of the 95% limit of agreement. The limit of agreement was calculated from differences between composite reference standard (CRS) and 18 individual ratings performed by nine raters on the set of 90 MAP bags. R1 to R5 were experienced raters, while R6 to R9 were inexperienced raters. Orange bars show frequency of overestimates, while blue bars show frequency of underestimates.

When individual raters were compared for the frequency of ratings that fell outside of the 95% limit of agreement, substantial differences were found between experienced and inexperienced raters (Fig 6). The frequencies of aberrant ratings for experienced raters ranged from 0.6% to 4.4% (mean = 2.1%), for inexperienced raters the frequencies were higher, ranging from 6.1% to 15.6% (mean = 9.4%). Two of the inexperienced raters (R7 and R8) mostly overestimated decay scores, while two other raters (R6 and R9) usually underestimated decay scores (Figs 4 and 6). This shortcoming in accuracy of ratings can be improved by extended training and instructions [16, 32], use of standard area diagrams [18], and continuing interaction with experienced raters.

### Timing of evaluations and combining of ratings

**Fig 7:**
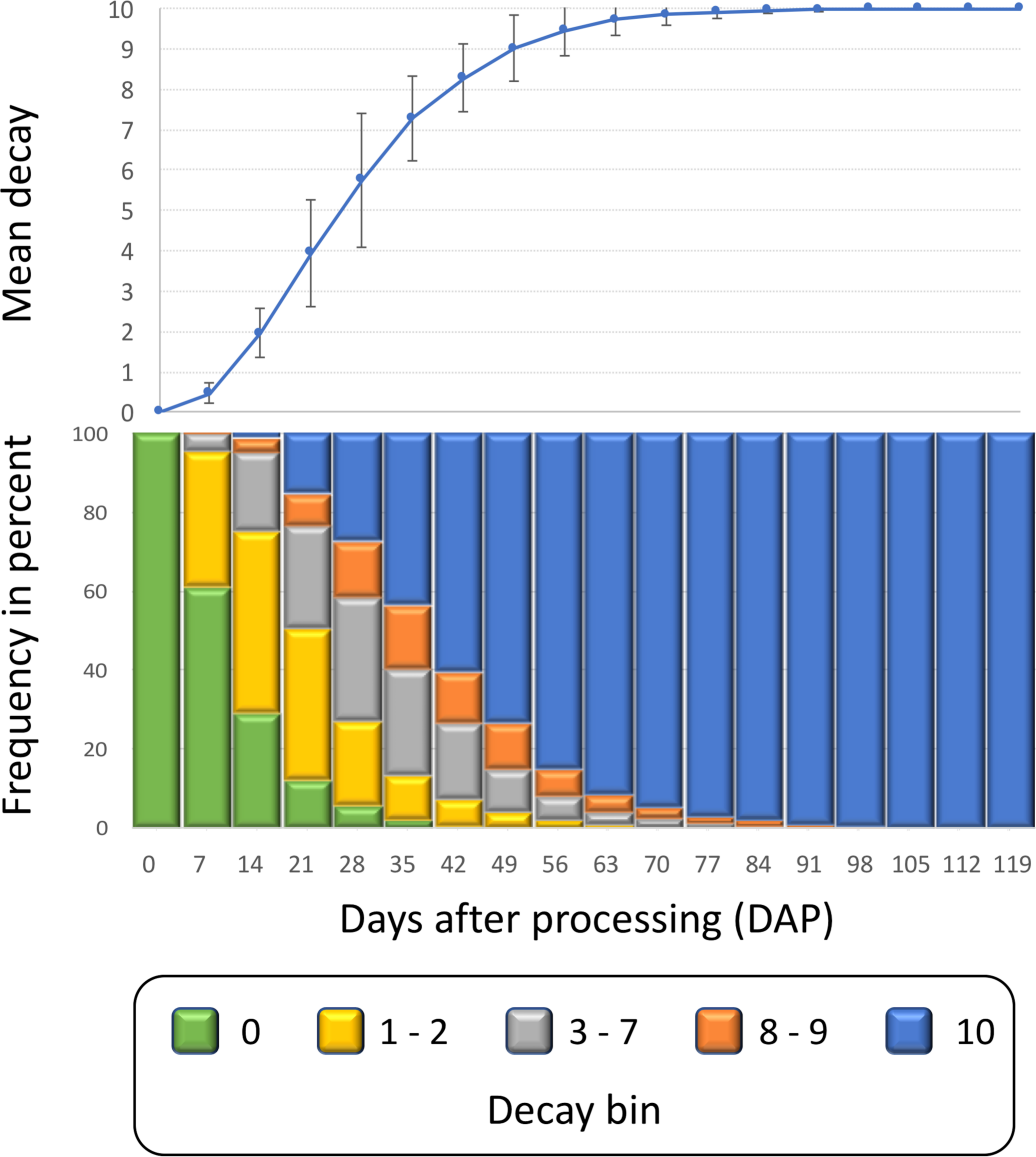
Decay progress calculated from 4,535 bags evaluated in eight different experiments. Upper panel shows the mean decay and the standard deviation of decay calculated from means of eight experiments. Lower panel shows the frequency of lettuce samples in five bins. These bins were developed for simpler presentation of data. They combine visual ratings from the 0 to 10 rating scale, where 0 indicates no decay and 10 indicates complete decay.

**Fig 8:**
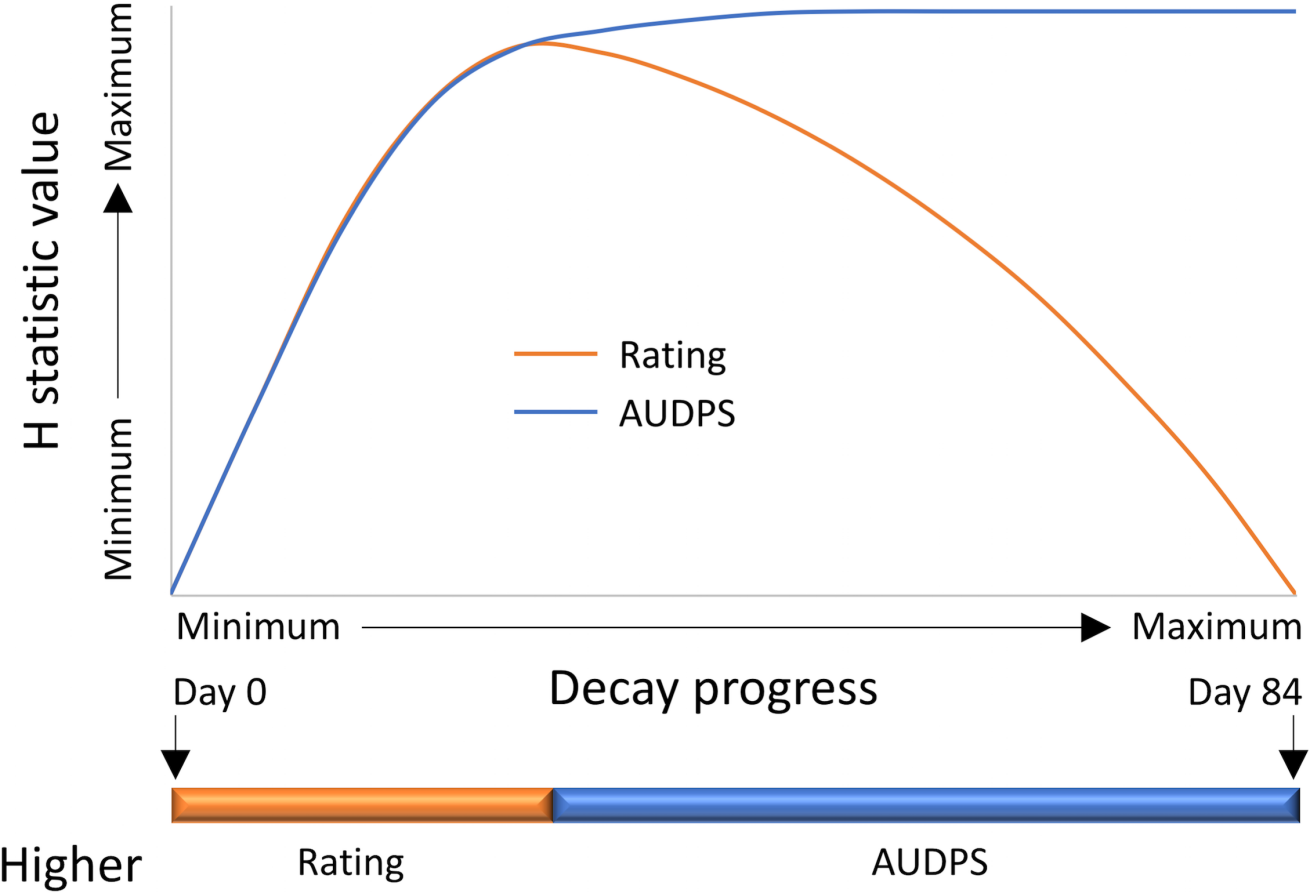
Profiles of H-values from Kruskal-Wallis tests that were calculated from either individual weekly ratings or from the area under the decay progress stairs (AUDePS) scores. H-values were calculated separately for eight experiments, scaled to the 0 to 100 scale (where 100 is the maximum H-value for the experiment) and averaged. The orange and blue horizontal line indicates periods where higher H-values were detected from ratings or AUDePS scores, respectively.

**Fig 9:**
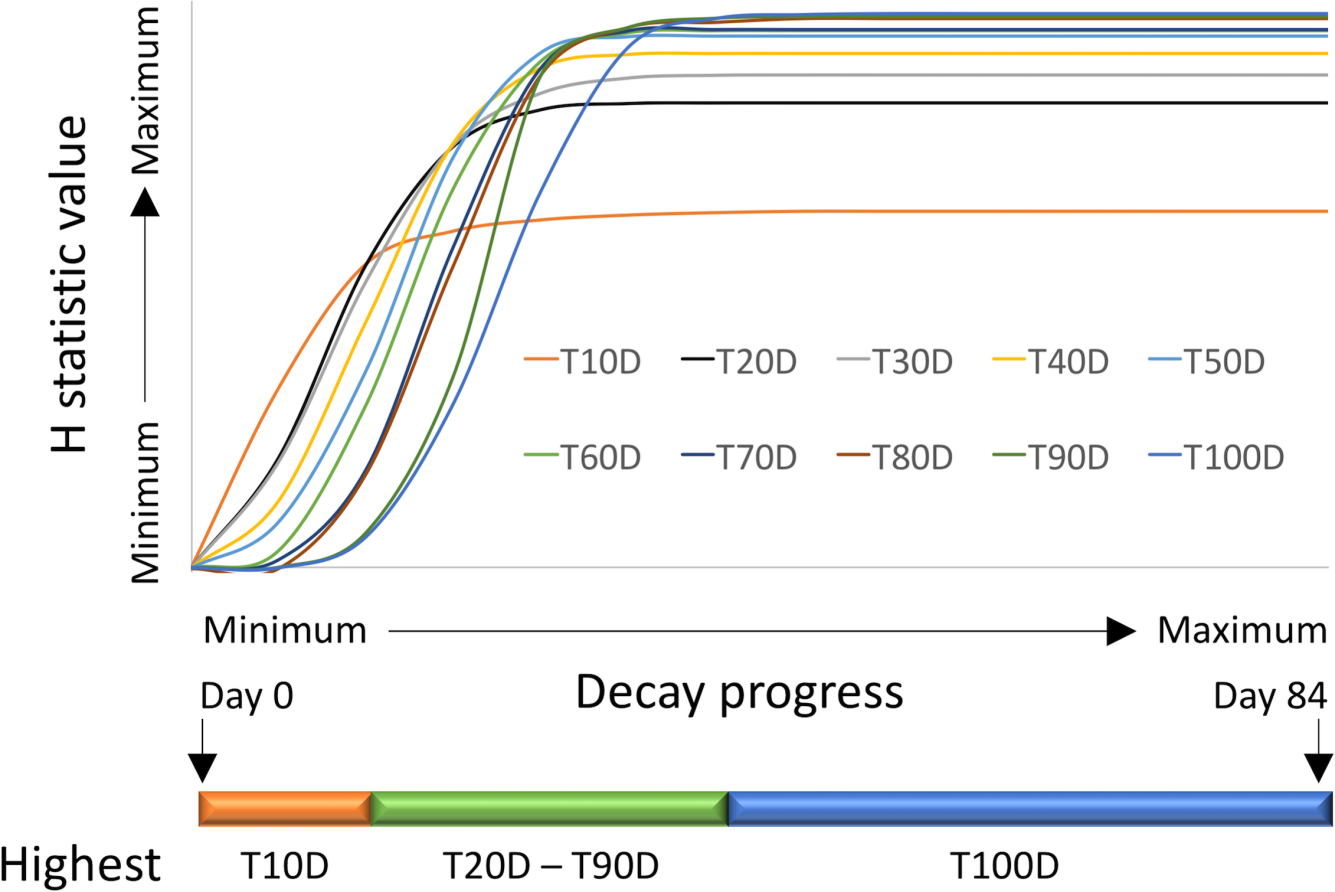
Profiles of H-values from Kruskal-Wallis tests that were calculated from estimates of time that is need to reach a certain level of decay (e.g. T10D is time to 10% decay, T100D is time to 100% decay). H-values were calculated separately for eight experiments, scaled to the 0 to 100 scale (where 100 is the maximum H-value for the experiment) and averaged. The orange, green, and blue horizontal line indicates periods where highest H-values were detected from T10D, T20D to T90D, or T100D, respectively.

Decay in fresh-cut lettuce is significantly affected by *qSL4*, an QTL that explained large portions of the phenotypic variation for decay in a bi-parental mapping population [6]. *qSL4* is known to be variable in our population and all our experiments included genotypes with both very rapid and very slow decay. Decay progress was similar in the eight analyzed experiments. The first signs of decay were observed in fast decaying genotypes within a week after processing (Fig 7). The fist rating of 10 (100% decay) was recorded 14 days after processing (DAP). An average decay rating of 5 was reached in all experiments around 21 DAP to 28 DAP. The last rating of zero (no decay) was recorded in a slow decaying genotype 42 DAP, and the last rating of less than 10 was recorded 112 DAP. KW tests performed on weekly ratings showed gradual increase in H-values until they peaked at the mean decay of approximately 5.5, about 28 DAP (Fig 8). When AUDePS scores were used for KW analyses, the H-values were almost identical to those from weekly ratings, until H-values from weekly ratings reached maximum. From that point, the H-values for ratings and AUDePS scores diverged. While those from weekly ratings declined until they reached zero, H-values calculated from AUDePS slightly increased and then plateaued. Since H-values using AUDePS plateaued with increasing numbers of assessments, rather than declined, multiple assessment time points combined into an index such as AUDePS will typically be preferred to analysis of a single assessment time points. When the KW tests were performed on data that estimated time to the certain level of decay (T10D to T100D), H-values differed substantially (Fig 9). H-values calculated from T10D grew fastest, were first to reach their maximum, but were also the smallest. Contrary, H-values calculated from T100D had a long lag period (because 100% decay is needed for the proper estimate of this score), peaked last, but were the largest. Difference between maximum H-values calculated from T100D, T90D, and T80D were, however, negligible. When maximum H-values calculated from AUDePS and T100D data were compared, in five out of eight analyzed experiments those calculated from AUDePS were higher. Comparison of these two parameters on 12 additional experiments revealed similar pattern, with the final ratio of 14:6 in favor of AUDePS scores. Using AUDePS may be preferred to T100D, because AUDePS scores take into consideration the whole decay progress including early stages of decay. AUDePS scores thus may find more subtle differences in decay than T100D scores. As described in the material and methods, all reported statistical analyses were performed with non-parametric KW test, because the distribution of some datasets was highly skewed, thus violating one of the assumptions for proper use of Analysis of variance (ANOVA).

The objective of this part of the project was to determine the best time for detecting significant differences among accessions. When ratings at only a single time point can be performed (due to labor or another constraints), the best time to find differences is approximately 21 DAP to 28 DAP, when average decay reaches about 50% (rating of 5). Analysis of earlier or later ratings may lead to less significant results (as indicated by lower H-values). If ratings have to be done at the specific time point (e.g. due to industry requirements) then the current analyses can indicate a relative size of H-value and related statistical significance. When assessments at multiple time points are performed, they can be combined into a single value such as AUDePS, or other calculated variables such as T100D. Based on our findings, AUDePS and T100D are expected to detect more significant differences than ratings at individual time points. It may not be desirable, however, to continue evaluations until tissue in all bags completely decays, even if H-values for AUDePS and T100D may possibly continue to increase. The increase in H-value is likely negligible. Our data indicate that it is not necessary to evaluate samples longer than 42 DAP when on average over 80% of tissue is already decayed. A minor increase in H-value may not justify additional use of valuable resources, such as labor for evaluations, or cold storage for keeping samples. In some instances, it may be desirable to know for how long the samples stay decay-free (fresh). This value is directly related to T10D. Therefore, to detect differences in longevity of freshness, we recommend evaluating samples frequently for up to 35 DAP. After this period, H-values plateau.

## Conclusions

The objectives of the present study were to compare the accuracy and the reliability of visual evaluations of decay on fresh-cut lettuce, and to determine the optimum timing for evaluations. Lin’s concordance coefficient (*ρ*_*c*_) that takes into consideration both the closeness of the data and the conformance to the identity line showed high repeatability (intra-rater reliability, *ρ*_*c*_ = 0.97), reproducibility (inter-rater reliability, *ρ*_*c*_ = 0.92), and accuracy (*ρ*_*c*_ = 0.96) for experienced raters. Inexperienced raters did not perform as well. Their under- or overestimates lead to decreased agreement between ratings, particularly reproducibility (*ρ*_*c*_ = 0.80) and accuracy (*ρ*_*c*_ = 0.90). Inaccurate ratings were more often observed in the middle of the rating scale than at the extreme ends. Therefore, we recommend that new raters receive training that includes practical examples in this range of decay, use of standard area diagrams, and continuing interaction with experienced raters (consultation during actual rating). Very high agreement among experienced raters indicate that visual ratings can be successfully used for evaluations of lettuce decay in MAP bags, until a more objective, rapid, and affordable method is developed.

For growing, processing and storing of samples we used procedures that imitate those used by the lettuce industry. Of course, multiple factors can nevertheless modify the rate of decay. This can be particularly problematic when using a single assessment time-point, as the mean amount and genetic variation for decay at any single time point can be dramatically different across experiments. To minimize the effects of production environment, the single-evaluation time-point can be based on a strategy that uses a pre-determined amount of decay in known check cultivars [13]. Such an approach may also minimize genotype × environment interactions that result from differences in genetic variation.

Prior to processing, evaluators should determine the general goal of the experiment. Many projects may seek to simply identify lines that are acceptable for commercial use, which are typically those that meet a minimum standard for performance. The longevity of freshness (or time to first decay) is typically of most interest in this case. Samples should be evaluated at multiple time points until 35 DAP (about 70% decay on average). Almost all of the material will likely start decaying by this time rendering further evaluations unnecessary. Breeding programs that seek to continually delay decay in successive breeding cycles will typically want to identify the genotypes with the slowest rate of decay, even among lines considered to be commercially acceptable. The best performing lines will be selected for advanced testing or use as parents to form new breeding populations. We recommend evaluating samples at multiple time points until 42 DAP (about 80% decay on average) and then combine these individual ratings into an AUDePS score.

Our results indicate fresh-cut decay experiments with weekly or more frequent evaluations that continue to approximately 42 DAP using trained evaluators can commonly detect significant difference among lettuce accessions. Applying this approach in populations that are genetically variable for decay should result, over time, in commercial cultivars with extended and more reliable shelf-life.

## Supporting information

S1 Dataset Decay scores on 90 bags of lettuce rated twice by nine raters. (XLSX)
